# *Streptomyces albireticuli* lung infection managed as a pulmonary air cyst: a case report and literature review

**DOI:** 10.3389/fcimb.2023.1296491

**Published:** 2024-01-11

**Authors:** Jiajiao Liu, Zhaoxia Xu, Yujie Bai, Jian Feng, Lunshan Xu, Fuxiang Li

**Affiliations:** ^1^Department of Neurosurgery, Daping Hospital, Army Medical University (Third Military Medical University), Chongqing, China; ^2^Department of Critical Care Medicine, The General Hospital of Western Theatre Command Chinese People's Liberation Army (PLA), Chengdu, China; ^3^Department of Emergency Department, The General Hospital of Western Theatre Command Chinese People's Liberation Army (PLA), Chengdu, China

**Keywords:** *Streptomyces albireticuli*, gene sequencing, lung infection, pulmonary air cyst, case report

## Abstract

*Streptomyces*, the largest genus in the *Streptomycetaceae* family and a prolific producer of antibacterial drugs, is a saprophytic soil organism that rarely causes invasive infections. Here we report a case of necrotic pneumonia caused by *Streptomyces albireticuli* in a 75-year-old man who presented with progressive chest tightness and dyspnea. *Streptomyces albireticuli* was isolated from his bronchoalveolar lavage fluid and identified through whole-genome sequencing (WGS) and phylogenetic analysis. The patient responded satisfactorily to clarithromycin therapy. The findings of this study may enhance our vigilance in identifying visceral infections caused by *Streptomyces*.

## Introduction

*Streptomycetes* are soil-dwelling microorganisms characterized by their gram-positive, filamentous, and branched forms (Barka et al., 2015). Known for their ability to produce rich and diverse secondary metabolites, they have been widely used in clinical medicine (Del Carratore et al., 2022). Despite the tremendous contributions of *Streptomyces* to medicine, they also pose risks as potential pathogens in humans, and particularly in immunocompromised patients (Herbrík et al., 2020; Gras et al., 2022). Among the documented cases of *Streptomyces* infection, only a few have been described as clinical isolates relevant to humans (McNeil et al., 1990; Gras et al., 2022). Actinomycetoma is the most commonly observed infection caused by *Streptomyces* spp. Additionally, *Streptomyces* can also cause rare invasive infections such as pulmonary infections and other diseases (Herbrík et al., 2020; Bai et al., 2021). A retrospective study of *Streptomyces* isolates identified from clinical samples in French microbiology laboratories showed that nearly half of *Streptomyces* infections with complete clinical documentation were invasive (Gras et al., 2022). Therefore, the significance of *Streptomyces* as opportunistic pathogens must not be overlooked. Herein, we present a case of severe pneumonia, further complicated by a pulmonary cavity infection caused by *Streptomyces albireticuli* in China. This report improves the identification and strengthens the diagnosis of this isolate.

## Case presentation

A 75-year-old man was admitted to our hospital experiencing chest tightness and dyspnea. His symptoms began ten days prior to admission, and included chest tightness, dyspnea, cough, and a small amount of brown sputum. His condition worsened over four days, prompting him to visit our hospital. He had sustained multiple fractures of the right ribs from a fall one month earlier and had applied unspecified herbs to the skin over the closed fractures. The patient had a history of smoking more than one pack of cigarettes per day for approximately 60 years.

**Figure 1:**
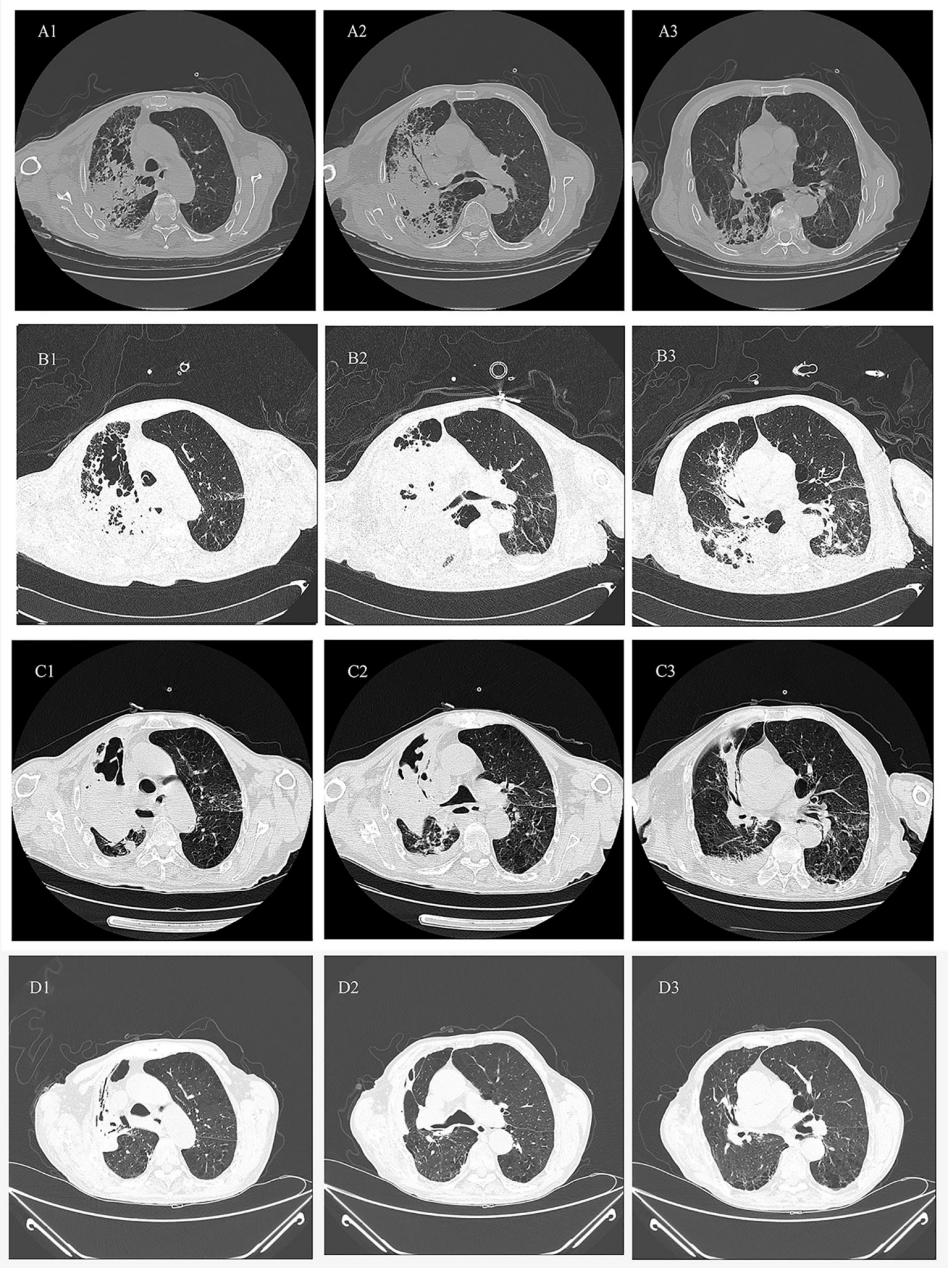
Series chest HRCT scans during his stay in the hospital and after discharge from the hospital. **(A1-A3)** scan obtained on illness days 10 showed patchy high-density shadows and a pulmonary air cyst in the right upper lobe. **(B1-B3)** scan obtained on illness days 17 showed the patchy shadows fused into lobe consolidation shadow and the pneumonia lesion and the air cyst apparently enlarged. **(C1-C3)** scan obtained on illness days 29 and **(D1-D3)** scan obtained 2 months after discharged showed the infected lesion in the right upper lobe and the volume of right lung gradually decreased.

On admission, he had a temperature of 36.7°C, blood pressure of 122/76 mmHg, pulse rate of 109/min, and a respiratory rate of 42/min. The patient’s oxygen saturation level was 88% in room air. He was conscious and showed scattered ecchymosis on the right neck, shoulder, and chest, with no palpable swelling of the superficial lymph nodes. Laboratory tests upon admission revealed elevated blood neutrophils (94.5%) and C-reactive protein (CRP) (283.91 mg/L) levels, while white blood cell (WBC) count and other parameters were normal. Chest computed tomography (CT) revealed patchy high-density shadows and a pulmonary air cyst in the right upper lung (**Figures 1A1–A3**). During hospitalization, the patient underwent endotracheal intubation and invasive mechanical ventilation due to respiratory failure. Bedside fibrobronchoscopy revealed a significant amount of brown purulent secretions in the right superior lobar bronchus, and bronchoalveolar lavage fluid (BALF) was collected. Considering the possibility of community-acquired pneumonia, intravenous piperacillin-tazobactam and levofloxacin were initially administered.

**Figure 2:**
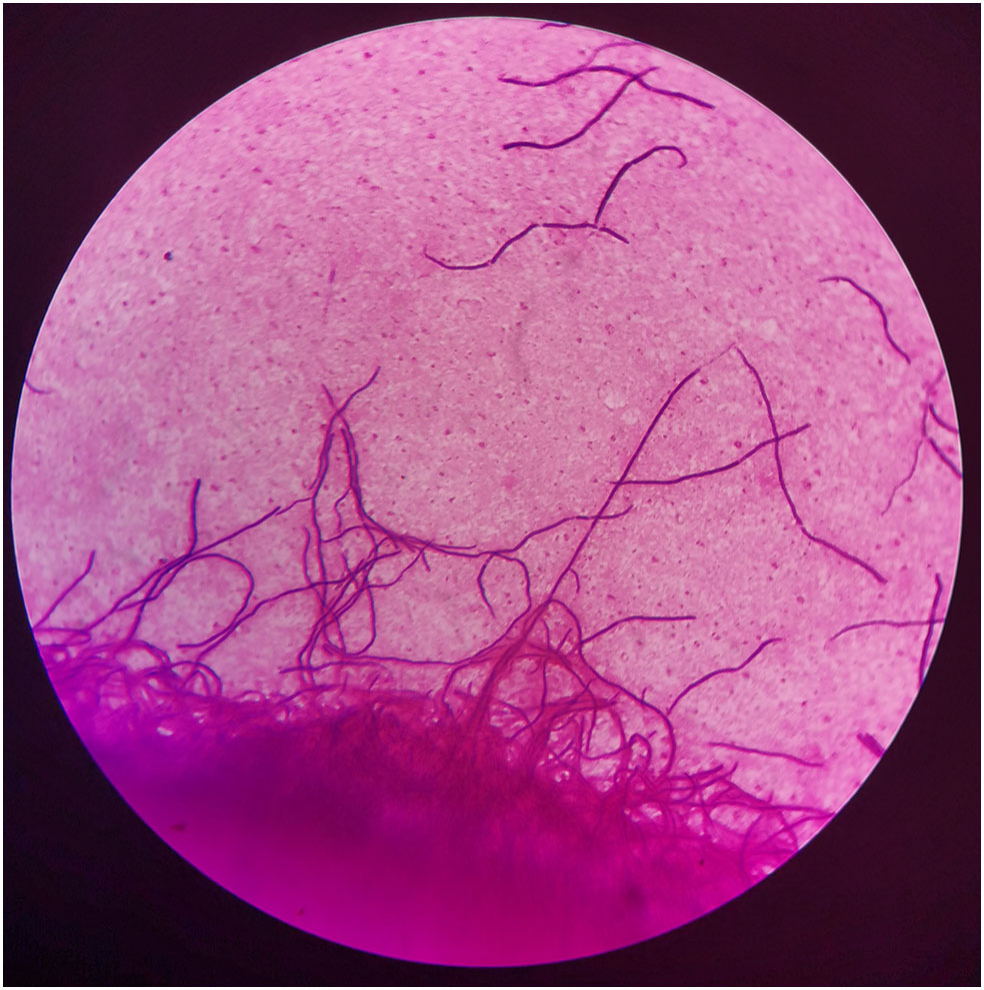
Gram stain of bronchoalveolar lavage fluids (BALF), demonstrating gram-positive, filamentous, nonspore-forming bacilli (×1000).

**Figure 3:**
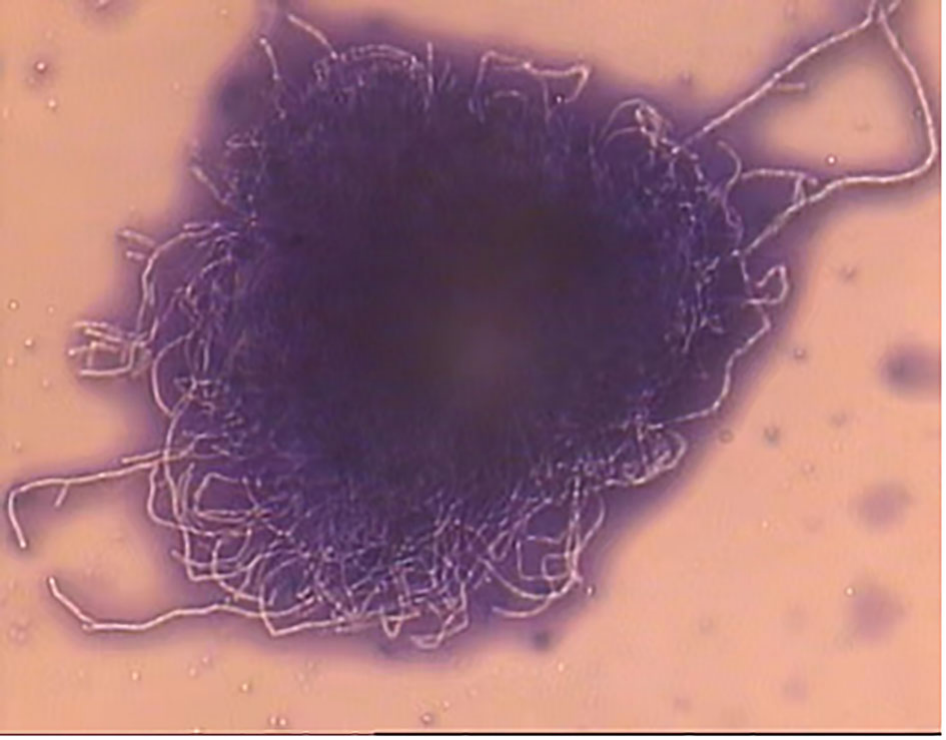
Laetophcnol cotton lalue stain stain demonstrating thin fast filaments (aerial hyphae) of *Streptomyces* spp. (×400).

**Figure 4:**
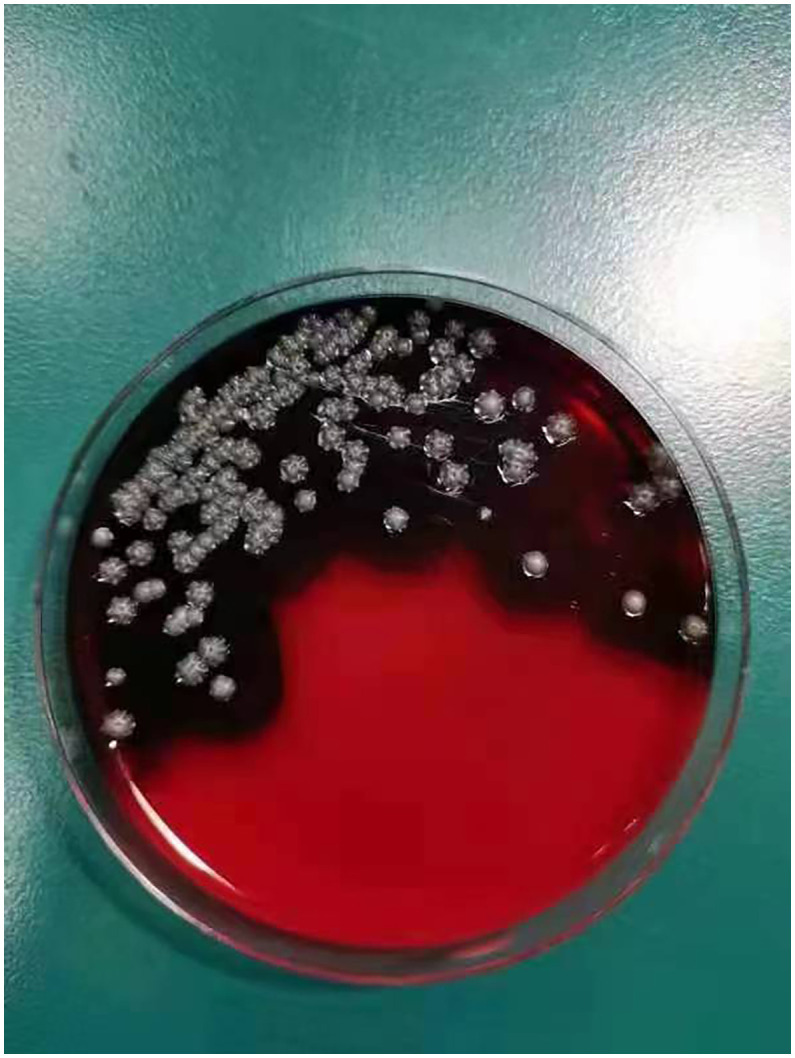
Blood agar appeared whitish, dry, with wrinkled small colonies biting agar.

On day +5 of his hospitalization, the BALF culture results showed gram-positive, branched, non-acid fast filamentous bacilli. These organisms developed aerial hyphae, which were clearly visible in the lactophenol cotton blue stain (**Figures 2**, **3**). The organism formed whitish, dry, wrinkled small colonies on blood agar, characteristic of *Streptomyces* species. These colonies exhibited velvety texture and developed white aerial hyphae after 72 h of aerobic growth (**Figure 4**). Initial empirical treatment (intravenous piperacillin-tazobactam and levofloxacin) was changed to linezolid and amikacin. Following significant improvement in the patient’s respiratory failure, he was extubated and provided with noninvasive ventilation via an oral-nasal mask. On day +8 of admission, an additional chest CT scan showed that the patchy shadows had fused into an enlarged lobe consolidation shadow, and a new left-sided pleural effusion was observed, indicating that the initial treatment was ineffective (**Figures 1B1–B3**). The organism was tested using the broth microdilution method to measure the minimal inhibitory concentration and was found to be susceptible to clarithromycin, amikacin, and linezolid (Kangtai Biotechnology Co., Ltd., Wenzhou). Clarithromycin was added, along with amikacin and linezolid, for 15 days. By day +20 of hospitalization, chest CT scans showed a gradual decrease in the consolidation shadow and volume of the right lung (**Figures 1C1–C3**). Upon discharge, the patient was instructed to continue clarithromycin therapy for two months. At a follow-up visit 2 months later, chest CT scans revealed that the lesion had been absorbed and both lungs had re-expanded (**Figures 1D1–D3**). There was no recurrence of the infection in the patient.

Considering the complexity of the diagnosis, we performed molecular identification using high-throughput sequencing to obtain the whole genome of the pathogen. DNA was extracted from the isolated strain and sequenced on an Illumina Nova-seq 6000 instrument (Illumina, San Diego, CA, USA) to produce 150-bp paired-end reads, at 100x coverage. High-quality reads were assembled *de novo* using MEGAHIT (version 1.2.5). The contig assembly base number was 9164494bp, the quality control parameter Q30 was 95.57%, and the GC percentage was 72.39%. The contig sequence had the highest similarity to the *Streptomyces albireticuli* MDJK11 sequence, with an average nucleotide similarity (ANI) value of 99.69%. The 16SrRNA was predicted using Barrnap (version 0.9) and was found to be similar to *Streptomyces albireticuli*, with 100% coverage and similarity. The sequence reads were deposited in the NCBI SRA database (accession numbers: PRJNA922082 [BioProject] and SAMN32642079 [BioSample]). A phylogenetic tree was drawn using BV-BRC, an information system combined with data and tools for bacterial and viral infectious diseases (Bacterial and Viral Bioinformatics Resource Center, version 3.28.9) (**Supplementary Figure 1**), using closely related *Streptomyces* species and different strains (published with complete genome sequence) of *Streptomyces albireticuli* from the National Center for Biotechnology Information (NCBI) database. Different strains of *Streptomyces albireticuli* clustered together, and the strain in this case clustered with *Streptomyces albireticuli* strain MDJK11.

The *Streptomyces albireticuli* strain possesses strong-hemolytic activity, which becomes apparent after 72h of cultivation, as shown in **Figure 4**. Hemolysis is considered a virulence factor in pathogens and is typically caused by the action of various protein hemolysins (Vandenesch et al., 2012). Alterations in the expression or deletion of certain virulence genes can lead to varying degrees of hemolytic activity (Zhu et al., 2017; Ravaioli et al., 2023). In our study, we compared the genetic sequence of our strain with genes previously reported to induce hemolysis (**Table 1**). The comparison parameters were set with a minimum coverage of 0.8 and a similarity threshold of 0.7. The analysis revealed that the 16S ribosomal RNA gene of *Brachyspira hyodysenteriae* strain G423 (MT304816.1) and the FilA5 gene of *Streptomyces filipinensis* strain DSM 40112 (MH638271.1) were comparable within the scope of this study. Thus, these genes may be responsible for the hemolytic activity of PRJNA922082.

**Table 1:** Putative hemolysin genes in the PRJNA922082 genome and alignment results.

Similar to	Matching results	GeneBank ID	Reference
*Brachyspira hyodysenteriae* strain G423 16S ribosomal RNA gene	match	MT304816.1	(Joerling et al.,2020)
Streptomyces filipinensis strain DSM 40112 FilA5 (filA5) gene	match	MH638271.1	(Herbrík et al., 2020)
Beta-Hemolysin (hlb) gene, e.g.,*Staphylococcus aureus* and *Staphylococcus pseudintermedius*	mismatch	JN580071.1	(Singh et al.,2014) (Kmieciak et al., 2016)
Hemolysin C (hlyC) gene,e.g.,*Escherichia coli*	mismatch	MN022628.1	(Juarez et al., 1984)
TlyC hemolysin of *Rickettsia prowazekii*	mismatch	CAA72456.1/Y11778.1	(Andersson and Andersson, 1997)

## Discussion

Streptomyces species, filamentous Gram-positive bacteria, represent the largest antibiotic-producing genus discovered to date (Del Carratore et al., 2022). Over 80% of clinically useful antibiotics and compounds are derived from *Streptomycetes* (Watve et al., 2001; Procópio et al., 2012). Commonly found in soil, *Streptomycetes* can infect the underground parts of living plants, causing scab disease in potatoes and affecting taproot crops such as carrots, beets, radishes, and parsnips (Bignell et al., 2014; Haq et al., 2023). However, the role of *Streptomycetes* as pathogens causing infectious diseases in humans has long been overlooked. In general, *Streptomycetes* cause suppurative granulomatous tissue changes in the skin (Relhan et al., 2017). Visceral infections are relatively rare, occurring mostly in patients with AIDS, advanced malignancies, or other serious diseases requiring immunosuppression (Kofteridis et al., 2007; Herbrík et al., 2020). Recent studies have confirmed the presence of *Streptomyces* in the respiratory tract, bloodstream, auditory canal and brain (Kapadia et al., 2007; Rose et al., 2008; Ai et al., 2020). However, only a few cases of *Streptomyces* pneumoniae in immunocompetent individuals have been reported to date (Kofteridis et al., 2007). Here, we describe a case of *Streptomyces albireticuli* pneumonia in an otherwise healthy man with no history of tuberculosis, chronic respiratory disease, or inborn immunodeficiency. However, it is important to consider that his immunity might have been compromised due to his advancing age and multiple rib fractures, which could predispose him to infection. As a farmer with a long history of exposure to soil and hay, he was at risk. *Streptomycetes*, being topsoil dwellers with spores significantly smaller than fungal spores, can easily reach the alveoli, posing a potential risk to individuals in such environments (Abdel Hameed et al., 1999). It should be noted that farmer’s lung disease is associated with the inhalation of *Streptomycetes* spores and fungal spores (Roussel et al., 2005; Cano-Jiménez et al., 2016). The patient lived in cool, damp conditions, environments where *Streptomycetes* are often cited as infectious agents in inflammatory diseases (Huttunen et al., 2003). These factors likely contributed to the colonization of human tissues by this *Streptomyces* strain, enabling it to become pathogenic.

*Streptomyces* infection is likely to be underdiagnosed, mainly due to a lack of awareness of its clinical relevance and the limited number of reports (Kofteridis et al., 2007; Herbrík et al., 2020; Kotrbová et al., 2022). Moreover, the definitive diagnosis of certain pathogenic infections requires a link between clinical manifestations and microbiological evidence (Siddig et al., 2022; Colom et al., 2023). The latter involves isolation of the pathogen from aseptic sources or direct microscopic identification of the infected tissue (Ataiekhorasgani et al., 2014). *Streptomyces* isolation in clinical cases is difficult due to its slow growth rate, whereas common respiratory microbiota grow more rapidly during routine cultivation (Kotrbová et al., 2022). The ubiquitous nature and low pathogenicity of *Streptomycetes* contribute to their controversial status when isolated alongside other primary pathogens. Additionally, *Streptomyces* infections are often underdiagnosed due to technical and methodological limitations. *Streptomyces-*specific polymerase chain reaction (PCR) targeting the 16S rRNA gene is not widely used in clinical microbiology laboratories. Although *Streptomyces* infections have been identified in the literature using 16S rRNA sequencing, 16S rRNA sequencing does not identify pathogens at the species level. Thus, multilocus sequence analysis (MLSA) is needed to accurately assign strains to specific species. Even with a similarity score above 99.6%, the 16S rRNA sequence alone is insufficient for species-level classification, necessitating the inclusion of a set of housekeeping genes in the analysis. In our case, 16S rRNA sequencing identified more than 10 species with more than 99% similarity, and most of them had only partial sequence data; therefore, it was difficult to identify them using housekeeping genes. Thanks to advancements in high-throughput sequencing technologies over the past two decades, whole-genome sequencing (WGS) can accurately identify strains, including isolate characterization, antimicrobial resistance genes, and virulence genes, showing great potential for clinical microbial diagnosis (Balloux et al., 2018). In our case, the pathogen was isolated from bronchoalveolar lavage fluid (BALF), avoiding contamination with other specimens, and was identified as *Streptomyces albireticuli* through WGS coupled with phylogenetic analysis. Additionally, no clinical or radiological improvements were observed after empirical antibiotic treatment. Hence, our diagnosis of *Streptomyces* as the primary pathogen was well established.

The clinical and imaging manifestations of *Streptomyces* pneumoniae infection reported in the literature are non-specific and diverse (Siddiqui et al., 2007; Riviere et al., 2012; Tiotiu et al., 2013). In this study, the patient presented with chest tightness, dyspnea, pulmonary air cysts, and pleural effusion on chest CT scans. However, the main results of antibiotic susceptibility testing (AST) for *Streptomyces* across different studies are generally consistent. Kotrbová et al. analyzed 84 *Streptomyces* clinical isolates to identify and evaluate their antibiotic susceptibility profiles. They found that *Streptomyces* species were most susceptible to amikacin, gentamycin, vancomycin, and linezolid, and exhibited high susceptibility to tetracycline and clarithromycin, while showing intrinsic resistance to penicillin (Kotrbová et al., 2022). Similarly, in two retrospective studies of human clinical samples, amikacin and linezolid covered 100% of the *Streptomyces* isolates (Sáez-Nieto et al., 2021; Gras et al., 2022). Consistent with these studies, our AST results showed that the pathogen was sensitive to clarithromycin, amikacin, and linezolid. *Streptomyces* is a slow-growing bacterium; therefore, extended antimicrobial therapies are necessary (Hamid, 2011). Treatment durations ranging from 6 to 24 weeks have been reported for most patients in the literature (Kofteridis et al., 2007; Datta et al., 2012; Riviere et al., 2012; Ataiekhorasgani et al., 2014). Our patient was initially administered broad-spectrum antibiotics and then switched to clarithromycin for nearly 3 months based on the sensitivity findings.

*Streptomyces* infections are rare and occur mainly in immunodeficient patients, thereby limiting clinical experience and management strategies. Specific antibiotic selection and treatment duration should be determined based on drug sensitivity results and clinical features of the patient.

## Conclusions

Herein, we describe a case of severe pneumonia caused by *Streptomyces albireticuli* in a male patient with normal immune function. After almost 3 months of clarithromycin treatment, the patient’s condition improved and the infection did not recur. WGS and phylogenetic analyses were used to identify *Streptomyces albireticuli*. The diagnosis and treatment of *Streptomyces* infection described in our case can provide a reference for the clinical management of such patients. For high-risk patients suspected of infection by rare pathogens, WGS testing is recommended when routine microbiological testing is inconclusive. Further researches are required to better understand predisposing factors, course, treatment, and evolution of *Streptomyces* isolates.

## Data availability statement

The original contributions presented in the study are included in the article/**Supplementary Material**. Further inquiries can be directed to the corresponding authors.

## Ethics statement

The studies involving humans were approved by Institutional Ethics Board of Western Theater General Hospital. The studies were conducted in accordance with the local legislation and institutional requirements. The participants provided their written informed consent to participate in this study. Written informed consent was obtained from the individual(s) for the publication of any potentially identifiable images or data included in this article.

## Author contributions

FL: Conceptualization, Data curation, Investigation, Methodology, Software, Supervision, Writing – review & editing. JL: Conceptualization, Investigation, Methodology, Software, Supervision, Writing – original draft. ZX: Investigation, Software, Writing – original draft. YB: Data curation, Supervision, Writing – original draft. JF: Validation, Visualization, Writing – review & editing. LX: Conceptualization, Methodology, Project administration, Supervision, Validation, Visualization, Writing – review & editing.
