# Supplementary material for: Streptomyces albireticuli lung infection managed as a pulmonary air cyst: a case report and literature review
Source: Front Cell Infect Microbiol. 2024 Jan 11;13:1296491. doi: 10.3389/fcimb.2023.1296491 (PMC10808341; doi:10.3389/fcimb.2023.1296491)
Supplement: Supplementary file 1 [file Image_1.pdf]

Supplementary Material

Streptomyces albireticuli lung infection managed as a pulmonary air cyst: A case report and literature review

Jiajiao Liu[1,2], Zhaoxia Xu[3], Yujie Bai[2], Jian Feng[2*], Lunshan Xu[1*] and Fuxiang Li[2*]

***Correspondence:**

Fuxiang Li, lfx98@163.com

Supplementary Figure 1

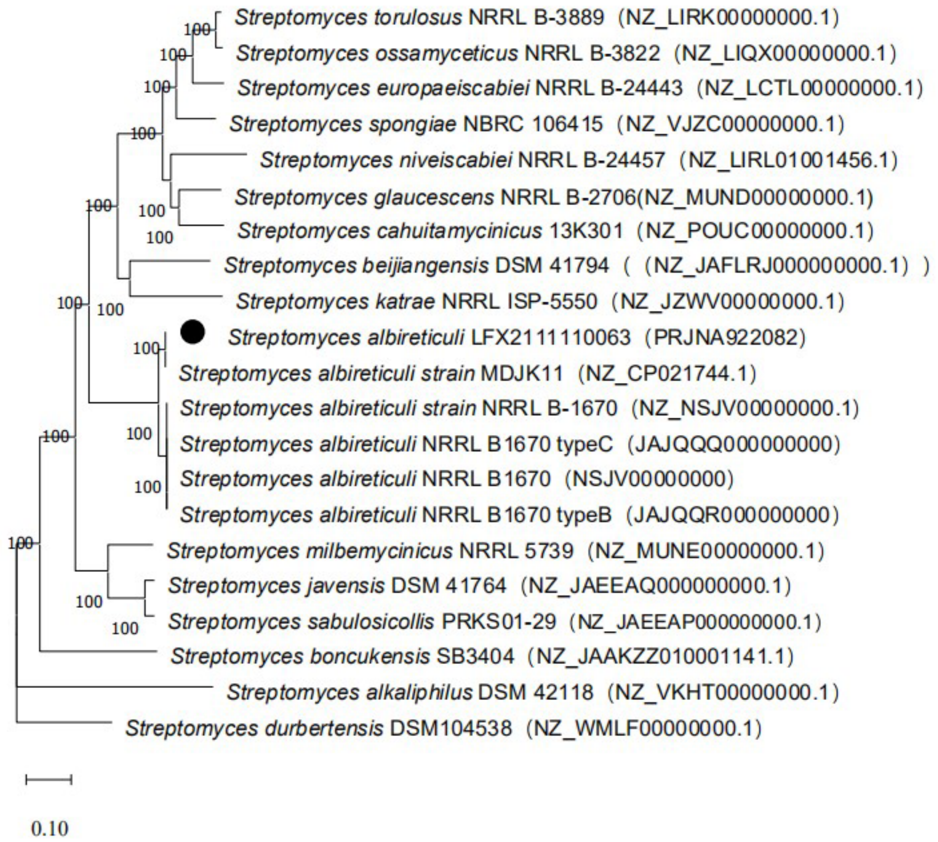

Supplementary Figure 1. Neighbor-joining phylogenetic tree of the genus Streptomyces albireticuli based on the complete genome sequence form NCBI.
